# Changes in the metabolome, lipidomein, and gut microbiota in Behçet’s disease

**DOI:** 10.3389/fcell.2025.1530996

**Published:** 2025-03-28

**Authors:** Chen Shang, Sujuan Ji, Wenting Hao, Xiangyu Wei, Jiani Yu, Jiayi Liu, Baoguo Zhang

**Affiliations:** ^1^ Department of Rheumatology and immunology, Xuzhou First People’s Hospital, The Affiliated Xuzhou Municipal Hospital of Xuzhou Medical University, Xuzhou, Jiangsu, China; ^2^ Department of Ophthalmology, The Affiliated Xuzhou Municipal Hospital of Xuzhou Medical University, Xuzhou First People’s Hospital, Institute of Eye Disease Prevention and Treatment of Xuzhou, Xuzhou, Jiangsu, China; ^3^ Jiangsu Key Laboratory of Immunity and Metabolism, Jiangsu International Laboratory of Immunity and Metabolism, Department of Pathogen Biology and Immunology, Xuzhou Medical University, Xuzhou, Jiangsu, China

**Keywords:** Behçet disease, metabolomics, gut microbiota, lipidomein, autoimmune illnesses

## Abstract

**Backgrounds:**

There is growing evidence that autoimmune illnesses are associated with the metabolome and microbiota. Because Behçet’s disease (BD) is not often diagnosed as a systemic disorder, the aim of this research was to investigate changes in gut flora and metabolites in BD patients.

**Methods:**

We used 16S rRNA gut microbiota gene sequencing and UPLC-QTOF-MS analysis to gather stool and serum samples from 12 age-matched healthy controls and 17 BD patients. The correlation between changes in gut microbiota and metabolites was then further analyzed.

**Results:**

In contrast to healthy controls, our investigation revealed significant changes in the makeup of gut flora in BD patients. In particular, we observed that in the BD group, there was a large drop in clostridia but a noticeable rise in γ-proteobacteria and betaproteobacteria. The serum metabolomics profiles of BD patients and healthy controls may be reliably differentiated using unsupervised principal component analysis (PCA). Several metabolites, including L-phenylalaine, tricarballylic acid, beta-leucine, ketoleucine, ascorbic acid, l-glutamic acid, l-malic acid, d-glucopyranuronic acid, and methyl acetoacetate, were found to have differential expression between BD patients and healthy controls. All of these metabolites were significantly lower in the BD group. Furthermore, we discovered strong associations between the detected metabolites such as tricarballylic acid, L-malic acid, D-glucopyranuronic acid with certain microbial families, such Prevotellaceae and Alcaligenaceae.

**Conclusion:**

Patients with BD were found to have significant changes in the makeup of their gut flora and metabolites.

## Introduction

Behçet’s disease (BD) is a widespread systemic inflammatory disease that mostly affects areas around the Mediterranean basins and the old Silk Road (Bulur and Onder, 2017). Hulusi Behçet, a Turkish dermatologist, first reported it in 1937 (Tugal-Tutkun, 2023). It is characterised by a variety of clinical symptoms, such as skin lesions, relapsing-remitting panuveitis with retinal vasculitis, gastrointestinal manifestations, arthritis, and vaginal and oral aphthous ulcers (Nieto and Alabau, 2020; Vaiopoulos et al., 2016; Ksiaa et al., 2019). The gastrointestinal symptoms caused by BD are very common in clinical practice (Emmi et al., 2024; Skef et al., 2015). Any portion of the gastrointestinal system might be affected by gastrointestinal symptoms, which are especially serious and entail a high risk of morbidity and mortality (Skef et al., 2015). However, the treatment effect is not good (Manuelyan et al., 2024).

The primary mechanical feature of BD is multi-organ involvement in inflammation. In comparison to people with recurrent aphthous stomatitis (RAS) and healthy controls, patients with BD have higher periodontal scores, and these values are connected with the severity of the disease (Ghate and Jorizzo, 1999; Jorizzo et al., 1985). The pathophysiology of BD is associated with infectious agents because to the molecular mimicry that occurs between human proteins and microbial antigens, specifically involving heat shock proteins (Gomes et al., 2018; Pay et al., 2007). Immunopathogenesis centers around T cells (Arayssi and Hamdan, 2004; Pineton de Chambrun et al., 2012), neutrophils, and antigen-presenting cells, with elevated levels of various inflammatory markers documented during active disease phases, providing insights into potential therapeutic targets (Musabak et al., 2006; Nuamah et al., 2023). As a result, it offers guidance for the inflammatory damage process and potential therapeutic targets.

The relationship between BD and gut microbiota has been preliminarily studied in previous studies (Yasar Bilge et al., 2020). The pathogenesis of BD may be due to a deficiency in immune tolerance due to a decrease in Tregs, while an increase in Th17 cells triggers inflammation. Recent studies have confirmed that changes in gut microbiota are involved in the development of BD by regulating Th1, Th17, and Treg cells (Ma et al., 2021). Consolandi and colleagues first reported the characterization of the gut microbiota in BD. Patients with Behçet showed significant depletion in Roseburia and Subdoligranulum spp., and the production levels of butyrate were significantly reduced compared to healthy controls (Consolandi et al., 2015). The intestinal flora of BD patients is rich in lactic acid-producing bacteria, sulfate-reducing bacteria, and some opportunistic pathogens, but lacks butyric acid-producing bacteria and methanogens (Chen and Tang, 2023; Sun et al., 2022). Alterations in microbial-derived metabolites represent an important mechanism by which changes in gut microbiota affect host health. For example, short-chain fatty acids (SCFAs), as a key set of metabolites produced by the gut microbiota, play a crucial role in bridging diet, gut microbiota, and host immune responses (Dupraz et al., 2021; Fan et al., 2023; Oezguen et al., 2019; Wang et al., 2023). Therefore, it may be of great significance to explore the changes of intestinal microbiota composition and metabolites in BD patients to explore the pathogenesis and treatment of BD diseases.

Through metabolite analysis, the developing field of metabolomics provides important insights into physiological and pathological states (Joshi et al., 2021; Karahalil, 2016; Reel et al., 2021). Metabolomic techniques have proven useful in the early identification of disease-related changes and have contributed to our knowledge of a number of disorders, such as diabetes, cancer, and autoimmune diseases (Wu et al., 2010), including cancer and diabetes (Xu et al., 2009). Although the metabolic anomalies in BD are complicated, there is mounting evidence linking the pathophysiology of non-communicable diseases (NCDs) to the gut microbiota (Lian et al., 2015; Di Lauro et al., 2023; Hamamah et al., 2022; Rinninella et al., 2020; Tan et al., 2023). Indirect microbial metabolism, molecular mimicry as a possible antigenic trigger, direct microbial translocation, or microbe-associated molecular patterns may all be the source of host-microbe crosstalk (Reinke et al., 2014; Zahoor et al., 2021). Studies on the pathophysiology of autoimmune disorders benefit greatly from the use of certain bacteria, and research on BD requires the establishment of relationships between metabolites and microorganisms (Guan et al., 2023; Belvoncikova et al., 2022; Christovich and Luo, 2022; Haneishi et al., 2023).

In this work, we analyzed the gut microbial profiles of BD using a variety of genomic techniques, such as 16S rRNA gut microbiota gene sequencing and UPLC-QTOF-MS. Our primary goals were to assess the relationships between microbial characteristics and metabolites, discover BD-specific features based on microbial metabolites, and examine microbial functions in BD patients.

## Materials and methods

### Patients

Between January 2010 and December 2023, the Xuzhou First People’s Hospital served as the single-center study’s enrollment site for 17 Behçet’s disease (BD) patients and 12 age- and sex-matched healthy controls who had no family history of autoimmune illnesses. Patients with BD fulfilled the revised International Criteria for Behçet’s Disease (ICBD) or the 1990 International Study Group criteria (International Team for the Revision of the International Criteria for Behçet's Disease, 2014; Blake et al., 2017). Data on the duration of the condition, clinical presentation, erythrocyte sedimentation rate (ESR), C-reactive protein (CRP) levels, and treatment regimens were gathered by clinical evaluations and a review of hospital records. Using the BD Current Activity Form 2006 (BDCAF 2006) (Author Anonymous, 1990; Kaklamani et al., 1998; Wong et al., 1984), disease activity was assessed. Patients were included when BD diagnosis was based on clinical, endoscopic, radiologic, and histopathological criteria established. Exclusion criteria were as follows: (i) Patients who were treated or were being treated for IBD prior to the study period; (ii) those who previously underwent gastrointestinal tract resection, which could cause nutrient deficiencies; (iii) patients with a history of cancer or with a stoma; (iv) patients lost to follow-up during the study period; and (v) those with incomplete medical records.

The Xuzhou First People’s Hospital ethics committee approved this research (Approval No.: XZFFD20180505, dated: 2018.10), and it adhered to the Declaration of Helsinki’s tenets. Written informed permission was acquired by each subject.

### 16s RNA sequence of gut microbiota

A faecal sample (1.0 g) was collected from each individual immediately after production. Several patients prepared the samples in their preferred locations and stored them at 4°C until they attended a hospital within 12 h of preparation. Each sample was suspended in 20% glycerol (Wako Pure Chemical Industries, Tokyo, Japan)/PBS and frozen in liquid nitrogen.

Samples were stored at −80°C until use. Deoxyribonucleic acid (DNA)was extracted and purified from the samples based on the literature with slight modifications (Liang et al., 2019).Briefly, after thawing, we filter the samples using a 100 μm mesh and wash them with PBS. Bacterial pellets were treated with lysozyme (Sigma-Aldrich Japan, Tokyo, Japan). The samples were then treated with achromatic peptidase (Wako Pure Chemical Industries). DNA was purified by SDS (Wako Pure Chemical Industries)/Proteinase K (Merck Japan, Tokyo, Japan) treatment, followed by phenol/chloroform extraction. After incubation with RNase A (Wako Pure Chemical Industries), sample DNA was precipitated with a polyethylene glycol solution (Wako Pure Chemical Industries). Samples are evaluated by measuring the ratio of optical density at 260 nm to optical density at 280 nm, typically 1.66 to 2.1. We then confirm the amplicon library using agarose gel electrophoresis (Shimizu et al., 2016).

After DNA extraction, the V4 region of the 16S rRNA gene was amplified using specific barcoded primers (V4F,5′-GTGTGYCAGCMGCCGCG GTAA-3′, and V4R, 5′-CCGGACTACNVGGG TWTCTAAT-3′). The PCR amplification product was mixed in equal amounts and measured by QuantiFluor. All samples were duplexed on the Illumina Hiseq PE250 (San Diego, CA, USA) platform. High-throughput sequencing analysis of bacterial rRNA genes using the Quantitative Insights into Microbial Ecology (QIIME, version 1.9.1) software suite. The calculated p-value is FDR corrected with FDR ≤0.05 as the threshold.

### Sample preparation for metabolomics/lipidomics analysis

Serum samples were obtained from blood samples after centrifugation at 1500 *g* for 10 min (4°C), alipacked, and stored at −80 °C for subsequent analysis. The sample pretreatment process is as follows: First, methanol (Fisher Scientific, Fair Lawn, USA) is added to 100 μL of serum and vortex to mix for 180 s. A total of 900 μL of methyl tert-butyl ether (Sigma-Aldrich, St. Louis, USA) and 250 μL of Milli-Q (Merck KGaA, Darmstadt, Germany) purified water were then added to the solution and vortex for 180 s. The mixture was then incubated on a rolling mixer for 10 min and kept at room temperature for 10 min before centrifugation at 13,000 × g for 10 min (4°C). A total of 700 μL of lipid extracts were transferred from the upper layer and 400 μL of polar metabolite extracts from the lower layer were transferred to two EP tubes, where they were concentrated and dried by vacuum centrifugation. The remaining samples were mixed and centrifuged, and the upper and lower layers with a similar distribution were used as quality control samples. Polar metabolite analysis was performed using three different analytical methods, and polar metabolite extracts were separated by reversed-phase chromatography to detect positive and negative ionization, respectively. Chromatographic separation of lipids is also performed in positive and negative ionization modes (Liu et al., 2024).

### UPLC-QTOF-MS analysis

Analysis of UPLC-QTOF-MS Parameters for mass spectrometry and chromatography were established as previously mentioned (Li et al., 2014). Positive and negative mode of an XEVO G2 QTOF was used for the mass spectrometry study. Accurate mass was maintained by introduction of LockSpray interface of sulfadimethoxine (311.0814 [M + H]+ or 309.0658 [M − H]−) at a concentration of 250 pg/μL in 50% aqueous ACN and a rate of 150 μL/min. The procedure included integration, normalization, and peak intensity alignment. In the positive data set, a list of m/z and retention time with corresponding intensities was provided for all metabolites in every sample. Then, the processed data set was then entered into the SIMCA‐P software package (v13.0, Umetric, Umea, Sweden). The normalized data were then used to perform principal component analysis (PCA) and orthogonal to partial least squares‐discriminate analysis (OPLS‐DA) with VIP >1 as a threshold. The chromatographic separation was performed on the Thermo Scientific Prelude SPLC system, and detection was performed on the Thermo TSQ Vantage triple quadrupole mass spectrometer (Guan et al., 2023). Statistically significant ions were putatively identified in MetaboLyzer, which utilizes the Human Metabolome Database (HMDB), LipidMaps, and the Kyoto Encyclopaedia of Genes and Genomes (KEGG) database. while accounting for possible adducts, H+, Na+, and NH4+ in the ESI + mode, and H–and Cl–in the ESI–mode (Author Anonymous, 1990; Kalra et al., 2014).

### Statistical analyses

Software called SPSS 17.0 was used to analyses the data, which were shown as mean ± standard error of the mean (SEM). The independent samples t-test, Wilcoxon rank sum test, or Mann-Whitney U test were used to evaluate group differences, and Spearman’s rank correlation coefficient was used to find correlations. For data analysis and visualization, GraphPad Prism v8.0 and R were used, with a statistical significance threshold of p < 0.05.

## Results

### Baseline

Our study included a total of 17 BD patients and 12 healthy volunteers. The mean age of BD patients was 34.25 ± 5.69 years, while that of the healthy volunteers was 35.91 ± 6.48 years (p = 0.36). The mean BMI of BD patients was 22.32 ± 3.62, while that of the healthy volunteers was 21.95 ± 2.25 (p = 0.158).

Among the BD patients, eight were males (47.06%), while there were five males in the healthy volunteer group (41.67%, p = 0.44). There were no statistically significant differences between the two groups in demographical and clinical characteristics such as smoker (p = 0.31), alcohol (p = 0.22), Type 2 DM(p = 0.12), hypertension (p = 0.06) and hyperlipidemia (p = 0.08). Detailed morbidity of BD patients is presented in Table 1.

**TABLE 1:** Demographical and clinical characteristics of patients with Behcet’s disease and normal individuals.

Characteristics at the time of sample collection	Behcet’s disease (BD, n = 17)	Normal individuals (NI, n = 12)
Age, mean years (range)	34.25 ± 5.69	35.91 ± 6.48
Men	8 (47.06)	5 (41.67)
BMI (Kg/m2)	22.32 ± 3.62	21.95 ± 2.25
Family history of BD	2	NA
smoker	8	3
Alcohol (Current)	3	2
Type 2 DM	5	2
Hypertension	4	1
Hyperlipidemia	3	1
Medication		
5-ASA	10	NA
Steroid	2	NA
Immunomodulators	5	NA
Biologics	4	NA
Disease activity parameters		
Oral aphthosis, %	17	0
Skin involvement, %	15	0
Genital ulcers, %	8	0
Uveitis, %	5	0
Gastrointestinal system involvement, %	4	0
Central nervous system involvement, %	2	0
Vascular involvement, %	2	0
Arthritis, %	2	0
Leucocyte	8.12 ± 0.89	NA
Neutrophil	5.44 ± 1.05	NA
IgG	11.84 ± 0.85	NA
IgA	2.61 ± 0.85	NA
IgM	1.31 ± 0.58	NA
IgE	68.25 ± 6.24	NA
Complement C3	1.09 ± 0.25	NA
CRP, mean ± SD, mg/dL	0.26 ± 0.11	NA

### Altered intestinal flora in patients with BD

**FIGURE 1:**
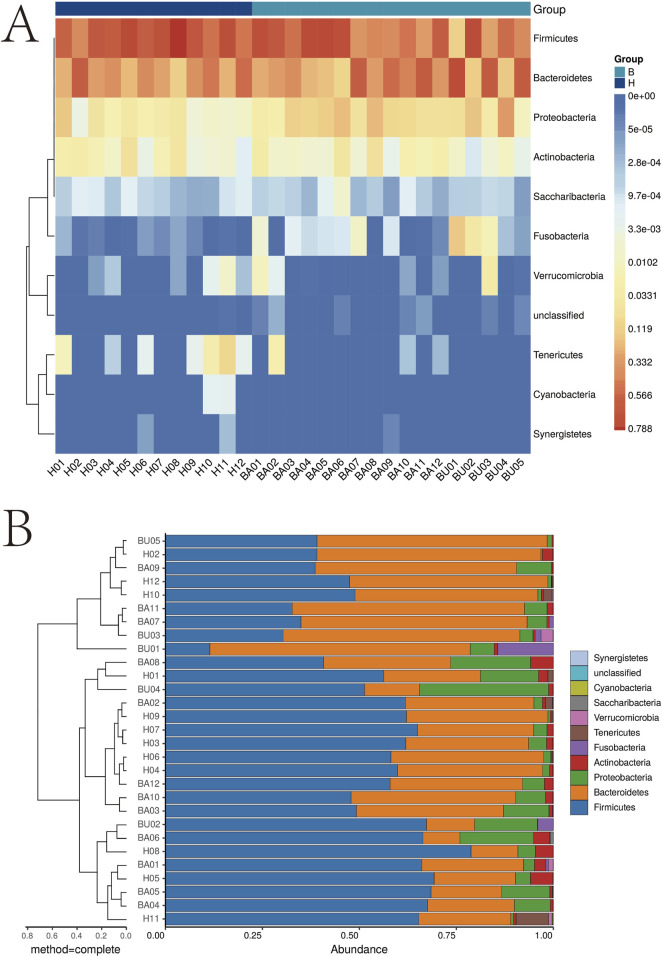
Cluster analysis of intestinal flora. *p < 0.05; **p < 0.01. **(A)** Intestinal microbiota analysis heat map; **(B)** Intestinal microbiota analysis dendrogram.

To investigate the impact of changes in intestinal flora and potential metabolites in Behçet’s disease (BD), we collected fecal samples from 17 BD patients and 12 healthy subjects. The samples underwent macro-genome sequencing, resulting in an average length of 6.23 ± 1.26 Gb per sample. We compared the obtained data with reference genomes available in the National Center for Biological Information (NCBI) and Human Microbiome Project (HMP) catalogs. Our findings revealed a significant increase in anamorphic and thick-walled bacilli, as well as a notable decrease in synergistetes and cyanobacteria. Heat map analysis and cluster analysis are presented in Figure 1.

**FIGURE 2:**
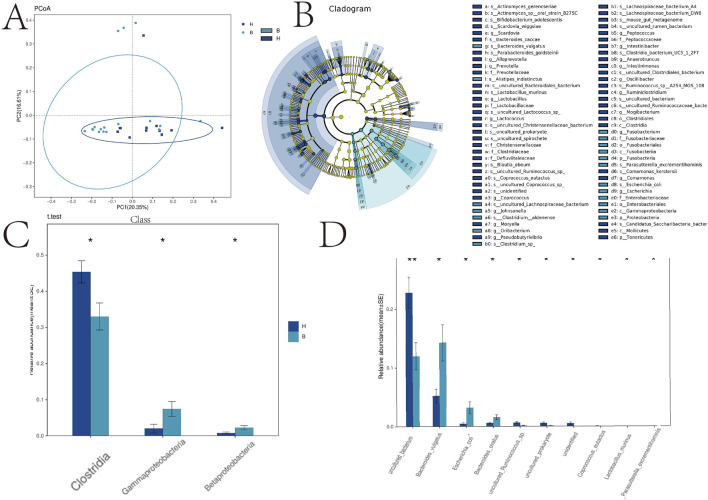
PCA analysis, taxa and species analysis of intestinal flora. *p < 0.05; **p < 0.01. **(A)** Principal Cause Analysis; **(B)** Caldogram map; **(C)** Distribution map of the main differential flora; **(D)** Distribution of phylum microflora.

In Figure 2A, we conducted a comprehensive causal analysis to identify significant alterations in gut flora between the two groups. A dendrogram illustrating the relationship between intestinal flora is presented in Figure 2B. Furthermore, we observed a significant decrease in clostridia but a significant increase in gammaproteobacteria and betaproteobacteria in the BD group compared to the control group (Figures 2C,D).

### Serum metabolomics

**FIGURE 3:**
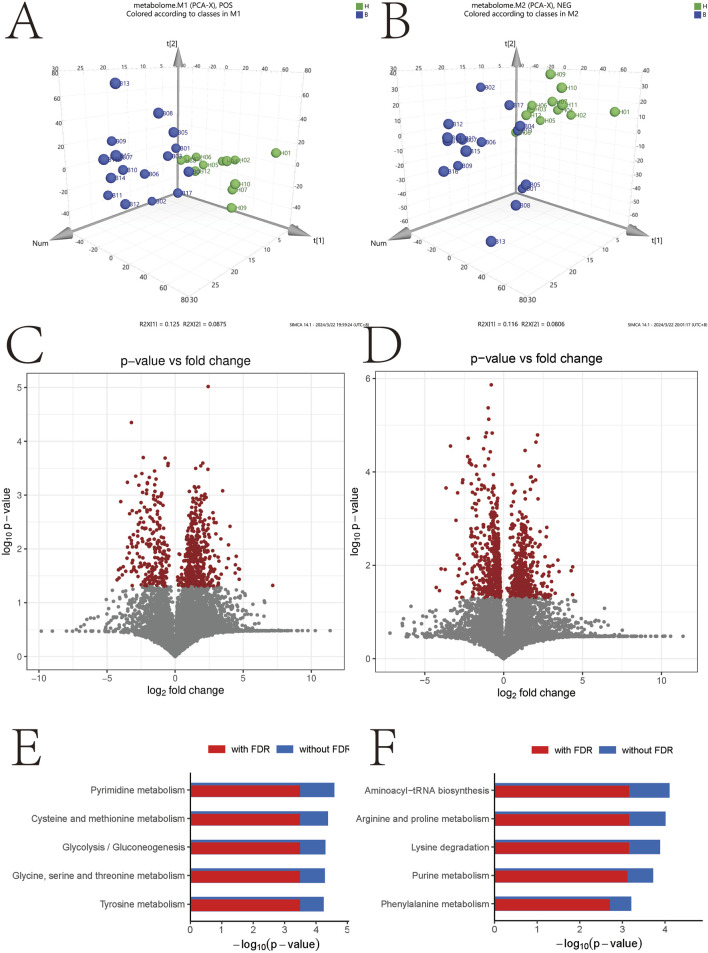
Negative- and positive-related volcano maps and signaling pathway analysis. *p < 0.05; **p < 0.01. **(A)** Positive molecular distribution map of metabolites in intestinal microbiota; **(B)** Distribution of negative metabolite molecules in intestinal microbiota; **(C)** Volcanograph of positive metabolite molecules; **(D)** Volcano map of metabolite-negative molecules, **(E)** Enrichment analysis of positive molecular signaling pathway; **(F)** Enrichment analysis of negative molecular signaling pathway.

To explore the metabolic profiles associated with BD, we performed metabolomics analysis on BD patients and healthy controls using the UPLC-QTOF-MS method. Unsupervised PCA plots were generated using SIMCA-P software, and differential analysis was conducted using Metabolic Analyzer. Volcano plots highlighting variations in ion levels between healthy controls and BD patients are depicted in Figure 3. In the volcano plot, red dots represent ions with significant differences (p-value <0.05) in levels between BD patients and healthy controls. Putative molecules for these differential ions were identified by cross-referencing metabolite databases based on precise mass numbers, as described in the Methods section.

**FIGURE 4:**
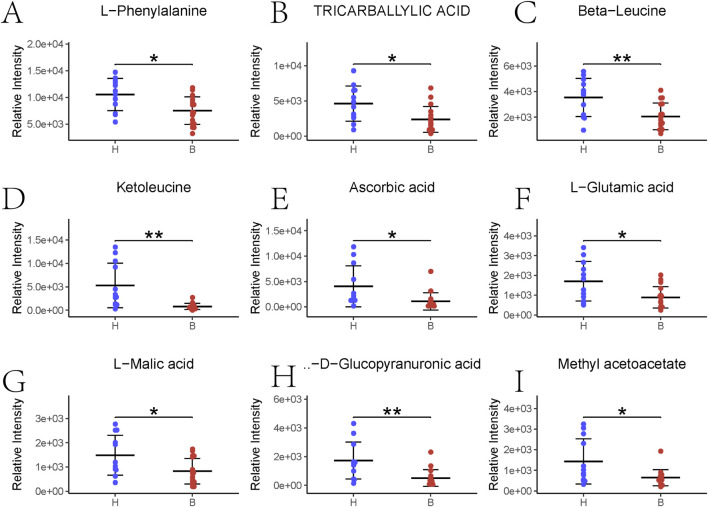
Top nine major gut flora metabolites sorted. *p < 0.05; **p < 0.01. **(A–L)** Phenylalanine abundance; **(B)** Tricarballylic acid abundance; **(C)** Beta-Leucine abundance; **(D)** Ketoleucine abundance; **(E)** Ascorbic acid abundance; **(F)** L-glutamic acid abundance; **(G)** L-malic acid; **(H)** D-glucopyranucronic acid abundance; **(I)** Methyl acetoacetate abundance.

Figures 3E,F illustrate the results of KEGG signaling pathway analysis, revealing a positive correlation between metabolic pathways associated with pyrimidine metabolism and cysteine and methionine metabolism, and a negative correlation with the aminoacyl-tRNA biosynthetic signaling pathway and arginine and proline metabolism. Noteworthy pathways with a false discovery rate (FDR)-corrected p-value <0.25 are highlighted. Supplementary Figure S1 displays heatmap results presenting the most significant alterations in nine metabolites. L-phenylalanine, tricarballylic acid, beta-leucine, ketoleucine, ascorbic acid, L-glutamic acid, L-malic acid, D-glucopyranuronic acid, and methyl acetoacetate exhibited a substantial decrease in the BD group compared to the control group (Figure 4).

### Metabolic pathways related with gut microbiota in BD

**FIGURE 5:**
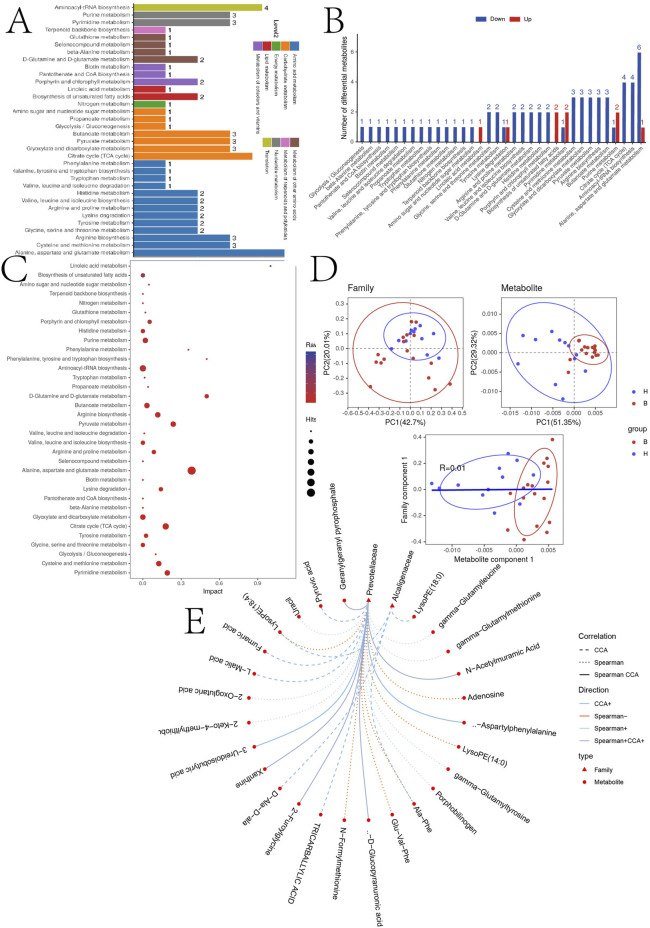
**(A)** KEGG pathway function annotation bar graph of positive ion compounds: the X-axis represents the number of metabolite annotations, and the Y-axis represents the annotated KEGG pathway. **(B)** Statistical upregulation and downregulation of pathway classification of differential metabolites. **(C)** Bubble plots for metabolic pathway enrichment analysis: X-axis enrichment factor (RichFactor) is the number of differential metabolites annotated to the pathway divided by identified metabolites annotated to the pathway. The larger the value, the greater the proportion of differential metabolites annotated to the pathway. The dot size represents the number of differential metabolites annotated to this pathway. **(D)** Scatter plot of correlation between differential metabolites and microbial groups: (1) The component scatter plot of microbial group; (2) The component scatter diagram of differential metabolite; (3) Pearson orrelation scatter diagram of the differential metabolite and the first component of microorganism group. The greater R is, the higher the degree of correlation between microorganism group and the first component of the metabolic pathway is. The color and ellipse represent sample groups. The greater the degree of sample dispersion in different groups, the better the classification effect of the component value. **(E)** Network diagram of correlation between differential metabolites and microbial groups at family level: the circle is the metabolite, the triangle is the microbial group; “−” represents negative correlation, “+” represents positive correlation.

Subsequently, we analyzed the signaling pathways for the expression of all cations and anions, summarized their effects on the host, classified and annotated the identified differential metabolites using the KEGG database, elucidated their functional characteristics, and identified major biochemical metabolic pathways and signaling pathways (Figures 5A–C).

To further explore the correlations between differentially expressed metabolites and the gut microbiota, correlation analyses were performed at the family level. Figure 5D demonstrates the sample categorization effects of the differential metabolites, as well as the correlation between the first component of the differential metabolites and the first component of the microorganisms. Strong Pearson correlation coefficients (R = 0.01) indicate a robust correlation between microbial taxa and the first component of the differential metabolites. The top 20 relationship pairs with the strongest ranked correlations and the top 20 relationship pairs with the strongest canonical correlations were combined and presented in a network diagram (Figure 5E), illustrating significant correlations of Prevotellaceae and Alcaligenaceae with the metabolites with tricarballylic acid, L-malic acid, D-glucopyranuronic acid.

## Discussion

Given the importance of maintaining microbiota balance for host health and the changes in gut microbiota observed during disease states, we investigated how microbial communities respond to disease perturbations. This makes sense. In our study, we observed significant changes in the intestinal microbiota and metabolites of patients with BD, especially those associated with changes in the families Prevotellaceae and Alcaligeneaceae.

Due to the absence of particular serologic markers, BD is a chronic autoimmune disorder with a variety of clinical presentations that may mimic other diseases. This makes it difficult to diagnose and treat the condition quickly. This study is the first attempt to identify putative biomarkers for BD diagnosis using metabolomics and intestinal microbiota techniques. Individuals with BD exhibited unique dysregulation of the gut flora, which included a notable decrease in butyric acid synthesis. Moreover, there was a notable decrease in Rhodobacter sphaeroides and Rose Bengal in the gut flora. A helpful metabolite of short-chain fatty acids, butyric acid influences immunomodulation and the mucosal immune response by promoting the growth of Tregs (Consolandi et al., 2015), and it is essential for maintaining the integrity of the intestinal epithelial barrier. Patients with BD had lower concentrations of the bacteria that produces short-chain fatty acids, *Clostridium difficile*, which led to lower levels of SCFAs and consequent immunological dysfunction (Shimizu et al., 2016). The abundance of Lachnospira and Barnesiellaceae was also found to be declining. While modulating the reduction in butyric acid production by influencing T cell differentiation and inflammation, Barnesiellaceae may have an anti-inflammatory effect by lowering Tumor necrosis factor alpha (TNF-α)levels, a critical cytokine in BD. On the other hand, Eggerthella levels were higher in BD patients, whereas Megamonas and Prevotella (van der Houwen et al., 2020) were less common. Lactobacilli were important for the BD microbiota, however *Bacillus* was consistently linked to increasing systemic inflammation (Shimizu et al., 2019). Our study also identified a range of changed gut flora in individuals with BD, including a significant drop in Synergistetes and Cyanobacteria and an increase in anamorphic and thick-walled bacilli.

Due to the absence of particular molecular indicators, early diagnosis and treatment of BD, a chronic autoimmune illness with a variety of clinical manifestations, are difficult. For this reason, identifying biological markers is essential to the diagnosis of BD. Based on prior study, L-phenylalanine, tricarballylic acid, beta-leucine, ketoleucine, ascorbic acid, L-glutamic acid, L-malic acid, D-glucopyranuronic acid, and methyl acetoacetate were shown to be important contributors in our inquiry. By boosting BH4 biosynthesis and decreasing superoxide generation by NO synthase, for example, L-phenylalanine’s antihypertensive actions may maintain renal and vascular function by lowering high ROS and NO levels (Wang et al., 2021). Notably, L-phenylalanine has promise in regulating inflammatory response and apoptotic signalling pathways, indicating that it may be a viable treatment option for prostate cancer (Zhang D. et al., 2023). The overexpressed LAT1 system absorbs L-phenylalanine quickly and with a high tumour selectivity. L-phenylalanine is a potential technique for oncologic SPECT imaging due to the availability of a kit and the tracer’s specificity (Kersemans et al., 2005). Since the 1960s, trans-aconitic acid has been associated with magnesium insufficiency in ruminants; new research indicates that rumen bacteria may be able to convert it into tricarballylic acid (Schwartz et al., 1988). According to research by Russell et al., rumen microbes produce tricarballylic acid, which may be harmful to ruminant tissue metabolism (Russell and Forsberg, 1986). A symmetric solid-state tricarballylic acid that is flexible and demonstrates optical transmittance and scalable areal capacitance (Choi et al., 2019). A flexible symmetric solid-state version of tricarballylic acid demonstrates optical transmittance and scalable areal capacitance. Additionally, tricarballylic acid has been tested for binding to the h-NK2 receptor and functional antagonist action on the bladder of rabbits (Harmat et al., 2002). Modifications to beta-leucine (homovaline) result in decreased hemolytic/cytotoxic effects, improved serum protease stability, and a little reduction in size (Verma et al., 2023). While leucine 2,3-aminomutase is known to occur in mammalian tissues, the presence of beta-leucine in human blood is not (Stabler et al., 1988). When compared to untreated denervated controls, animals treated with ketoleucine did not exhibit a statistically significant decrease in muscle wasting. Furthermore, animals given ketoleucine did not see a reduction in the excretion of 3-methylhistidine in their urine, which might be a sign of muscle breakdown (Yee et al., 1988). Biocompatible polymeric nanoparticles of glutamic acid may find use as a medication delivery system (Bakan et al., 2023). In a SOCS2-dependent way, L-malic acid polarises M2 macrophages and raises interleukin-10 levels (Zhang FL. et al., 2023). Alone or in combination, L-malic acid may reduce and inhibit oxidative damage brought on by CPF (Salyha and Salyha, 2018).

Our study also found that gut microbiota and metabolite correlation play an important role in metabolic diseases. Gut microbiota, including Prevotellaceae, Rikenellaceae and Ruminococcaceae and their metabolites SCFAs play important roles in intestinal barrier integrity and intestinal homeostasis (Wang et al., 2020). The gut microbiota Prevotellaceae family is capable of producing butyrate, and long-term treatment with nicotinamide mononucleotide (NMN) helps maintain gut homeostasis by modulating the gut microbiota (Huang et al., 2021). Prevotellaceae produces butyrate to alleviate PD-1/PD-L1 inhibitor-related cardiotoxicity via PPARα-CYP4X1 axis in colonic macrophages (Chen et al., 2022). Maternal l-malic acid consumption reshapes the colonic microbiota of its offspring. In short, the abundance of Colidextribacter, Romboutsia, and Family_XIII_AD3011_group increased, which was positively correlated with antioxidant capacity and glucose metabolism in skeletal muscle. Decreased abundance of Prevotella, Blautia, Prevotellaceae_NK3B31_group, and Collinsella were also detected, which were associated with lower insulin sensitivity (Zhang P. et al., 2023). We hypothesize that specific gut microbiota may play a key role in the pathogenesis of BD through its associated metabolites. We hypothesize that specific gut microbiota may play a key role in the pathogenesis of BD through its associated metabolites. However, there are still few studies on the association between other specific gut microbiota and metabolites, and further analysis is needed in future studies.

### Limitations

From the discussion above, it is clear that BD may affect gut flora and metabolites in a number of ways, including changes in the ratios of gut flora that are mediated by the neurological and psychological systems. By controlling metabolism and hormones, BD modifies alterations in gut flora and metabolites. It also mediates immune system and host inflammatory responses. Our research does, however, have certain shortcomings. Considering the complexity of the composition of the intestinal microbiota and the diversity of the composition of metabolites, although a variety of statistical methods were used for data analysis in our study, there were still many factors such as small sample size and short study time, so it is necessary to supplement the sample size and increase the long-term follow-up time in future studies to further clarify the impact of intestinal microbiota and metabolites on BD disease. Although we limited the type of treatment received to patients with BD in the inclusion and exclusion criteria, and the baseline data also included this part of the data, there may still be some impact on the results, and the sample size may need to be further expanded in future studies to ensure that patients with BD who received the same treatment are statistically analyzed. Moreover, BD has a number of side effects, including metabolic syndrome, which has been connected to gut flora. Consequently, further research is needed to examine these correlations. Further clinical research is necessary to confirm and build upon these results. Further investigation into the precise processes by which BD affects intestinal flora and maybe causes pathological alterations in other systems is necessary, and this will be the main goal of our next studies.

## Conclusion

Our research sheds light on the altered variety and abundance of gut metabolites and gut flora in BD patients, offering more understanding of the illness. It is still unknown, nevertheless, how gut flora and BD are causally related. Thus, further study is needed to look at possible processes and causal relationships between gut flora and BD.

## Data Availability

The original contributions presented in the study are included in the article/Supplementary Material, further inquiries can be directed to the corresponding authors.
