# Supplementary figures and images for: Changes in the metabolome, lipidomein, and gut microbiota in Behçet’s disease
Source: Front Cell Dev Biol. 2025 Mar 28;13:1530996. doi: 10.3389/fcell.2025.1530996 (PMC11997388; doi:10.3389/fcell.2025.1530996)

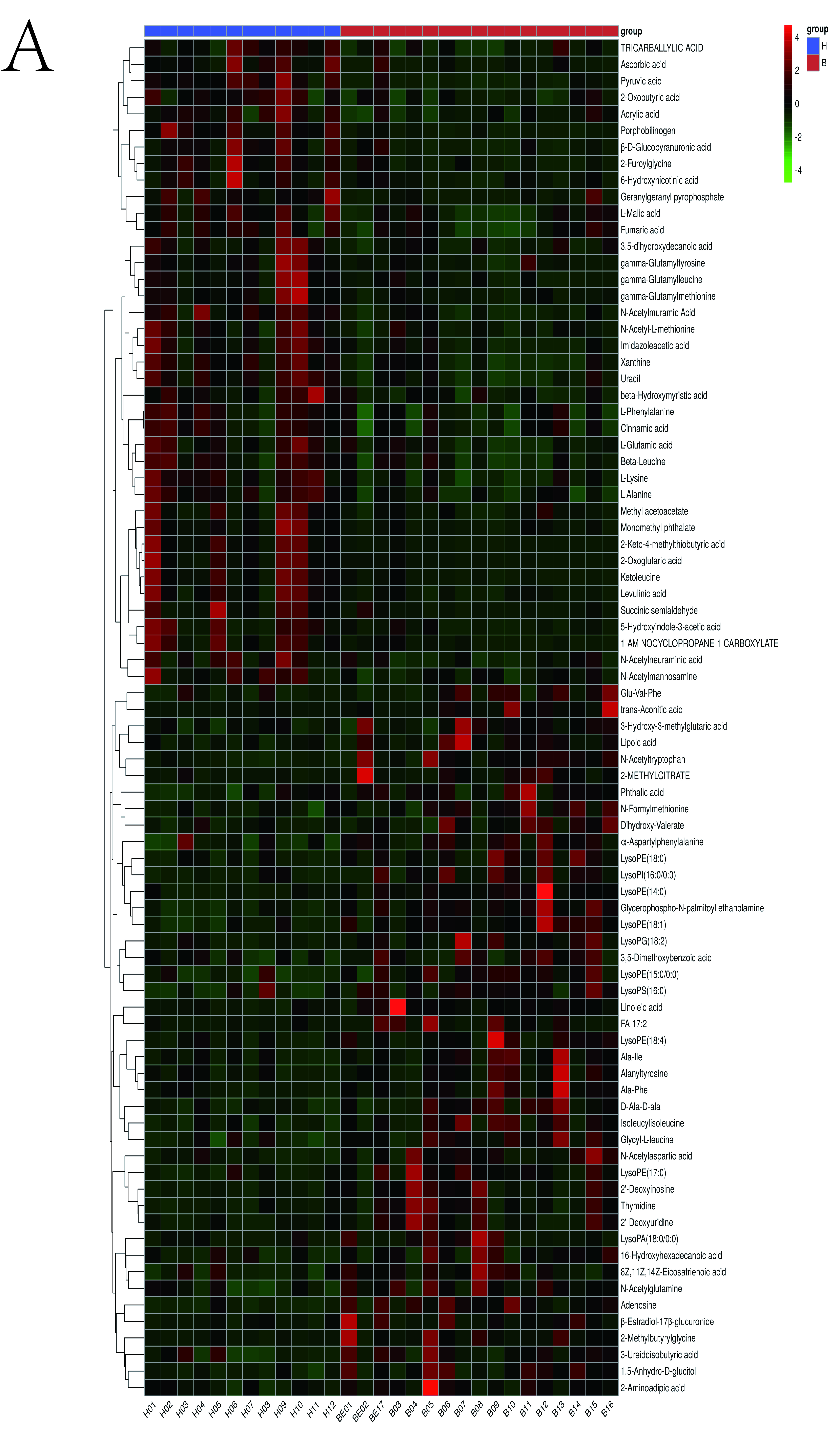

Supplement: Supplementary file 1 [file Image1.jpeg]
